# Thinking ethical and regulatory frameworks in medicine from the perspective of solidarity on both sides of the Atlantic

**DOI:** 10.1007/s11017-016-9390-8

**Published:** 2016-12-08

**Authors:** Barbara Prainsack, Alena Buyx

**Affiliations:** 1 Department of Global Health & Social Medicine, King’s College London, Strand Campus, 2.10 East Wing, King’s Building, London, WC2R 2LS UK; 2 Institute of Experimental Medicine, Christian-Albrechts-Universität zu Kiel, Arnold-Heller-Str. 3, Haus 28, 24105 Kiel, Germany

**Keywords:** Solidarity, Personal data use, Harm mitigation fund, Health policy, Data Protection Regulation

## Abstract

This article provides a concise overview of the history of scholarship on solidarity in Europe and North America. While recent decades have seen an increase in conceptual and scholarly interest in solidarity in North America and other parts of the Anglo-Saxon world, the concept is much more strongly anchored in Europe. Continental European politics in particular have given rise to two of the most influential traditions of solidarity, namely, socialism and Christian ethics. Solidarity has also guided important public instruments and institutions in Europe (e.g., welfare, healthcare, etc.). Despite the much stronger affinity of continental European societies to solidaristic thinking, we argue that solidarity has much to offer for addressing societal challenges on both sides of the Atlantic and beyond. After proposing a working definition of solidarity that highlights its utility for guiding policy and practice, we give an example of how a solidarity-based perspective can shape instruments for the governance of data use.

## Introduction: the roots of solidarity

Solidarity is gaining currency. Against the background of global economic crises, climate change, and violent conflicts, calls for solidarity are becoming louder and more frequent. Appeals to global solidarity, solidarity with refugees, or solidarity in protecting our environment play more important roles in theory and practice.

But what is solidarity? The scholarship on solidarity is diverse, and so are contemporary understandings of the term. Solidarity is used in different contexts, to support different goals, and with many different meanings. The one thing that most works on solidarity have in common is that they treat solidarity as something pro-social. Solidarity is portrayed as contributing positively to the social fabric of society, or enhancing social cohesion. Beyond this small common denominator there is no uniformity in how the notion of solidarity is used. Some authors see solidarity as an emotion [1], others as a moral ideal [2], as a ‘natural’ characteristic of groups or societies [3], or as a political idea [4]. Still others see it as an empty label, and some regard it as an attack on individual autonomy [5].

One reason for the under-determination of the notion of solidarity is the concept’s patterned history in Western thought, without the continuity of debate and systematic development that other terms have had, such as justice, responsibility, freedom, or liberty. One of the earliest uses of the term solidarity was within Roman law, where the phrase *in solidum* referred to something that was owed by more than one person, or owed to two or more people who were joint creditors [3, 6]. An explicit understanding of the term emerged only in the early 18th century with the increasing use and currency of *solidarité* during the French Revolution. From there, the idea of solidarity spread beyond the borders of France, especially into Germany and England, where it was taken up prominently by socialist and workers’ movements [7]. The idea of solidarity also underpinned early trade union developments and has played an important role in Marxist and socialist rhetoric to achieve mutual support amongst all workers, often with the distinguishing feature of disregarding national borders.

The work of one of the ‘fathers of sociology’, Auguste Comte, greatly increased the visibility of the term ‘solidarity’ and solidified its importance in the wider field of the social sciences. He envisaged solidarity as a remedy for the increasing individualisation and atomisation of society [8]. Comte’s work on solidarity has influenced most of the scholarship on solidarity after him, including Émile Durkheim, whose distinction between mechanical and organic solidarity is still one of the standard reference points within scholarship on solidarity [9].

Another important field contributing to the early development of solidaristic ideas and uses of the term was religious thought. The Catholic notion of solidarity, for example, with its strong roots in Thomas Aquinas’s work, emphasised community and fellowship between all human beings. This type of solidarity captured an important element that continues to influence contemporary understandings of solidarity, namely, the idea of fellowship of people who recognise relevant similarities between one another. It sets solidarity apart from charity, for example, where action is based upon differences between people and not on what they see as binding them together. In other words, charity is based on an asymmetrical relationship between those who are in need and those who have enough resources to give something to the former. Although there are different nuances to the specific meaning of solidarity in different Christian traditions, and additional ones in non-Christian faiths [10–13], in all religious traditions, the notion of solidarity is connected to discussions of social justice, the fellowship of all beings (or all believers) created in the image of God, and assistance to those in need. In other words, in religious contexts solidarity takes the role of a God given moral imperative to assist others in their quests for social justice, and more generally, for leading a good life.

This very short historical overview highlights several important elements of the meaning of solidarity that appear, in different guises, in each of the contexts and traditions. They are also still relevant today. These are: a sense of being bound together, e.g., by sharing similar characteristics or circumstances; mutual assistance and help, particularly in situations of hardships; symmetric relationships between those engaged in solidaristic practices at the moment of enacting solidarity (i.e., despite other aspects of their lives not being equal or even similar); and a link to personal and collective wellbeing.

In the remainder of the article, we consider the discussions of solidarity in continental Europe and North America respectively. We argue that the marginal role that solidarity has played in North American theory and politics is due to the political history of the continent, and—in the 20th century—to the association of the term with a particular party politics that was diametrically opposed to the liberal rhetoric in North America. We then introduce our own working definition of solidarity which, we argue, enhances the utility of the term for empirical analysis and policy making, and at the same time, frees the concept from its partisan political connotation (without rendering it apolitical). To illustrate that such an understanding of solidarity can indeed help to guide policy and practice, we present a governance framework for data governance that was developed on the basis of a solidaristic framework. A solidarity-based approach, we argue, has much to offer on both sides of the Atlantic, and in fact, globally. In times of economic, political, and ecological crisis, solidarity is needed more than ever.

## Solidarity in continental Europe

As has become apparent in the previous section, the concept of solidarity is rooted in Europe [7, 14]. First of all, solidarity has a distinctly European heritage in the sense that both its Christian and its socialist legacies emerged from social and historical configurations in Europe from the 18th century onwards. Some ethicists and theorists in fact continue to see solidarity most strongly anchored in Europe [15–20]. One of the most explicit voices is Hans-Martin Sass, who argued:

The principle of solidarity is a notion that is not easy for those in the Anglo-American philosophical tradition to understand. A richer notion than either social or legal reciprocity among freely contracting individuals, it is both presupposed by the sphere of self-interested social interaction, because it is a personal virtue, and complementary of that sphere, insofar as it is a principle of social morality, justifying institutions of social justice and welfare. [21, pp. 367–368]

Indeed, solidarity has played a larger and more explicit role in the institutionalisation of health and social care within Europe than outside. It is also often seen as characteristic of a particularly European understanding of welfare provided by public actors in the 20th century [22–24].

Matti Häyry, for example, also regards both precaution and solidarity as particularly (continental) European values vis-à-vis the ‘American “autonomy and justice” approach, which is often seen as overemphasising the role of individuals as consumers of health services’ [17, p. 199]. For him, other important differences between European and North American bioethics are, first, that Europeans tend to cherish additional values that go beyond ‘hedonism and egoism’, and second, that seemingly shared values such as autonomy, justice, beneficence, and non-maleficence are understood and enacted in different ways [16].

In contrast, discussing the four seminal principles from Beauchamp and Childress’s *Principles of Biomedical Ethics* [25], among others, Floris Tomasini warns of ‘spending too much time defining our principles in opposition to the Americans’ [20, p. 4].^1^ This, he argues, could put Europeans ‘in danger of looking insecure and creating a “resistance identity”,^2^ further legitimating the orthodoxy of the four principles’ [20, p. 4]. Tomasini then puts forward a set of values (not principles) that he considers particularly European: inclusivity and scope, particularity and context, multiplicity and rigor, deep questioning, harmony, consensus and disagreement, and pluralism [20, pp. 5–6].

## Solidarity in the Anglo Saxon world

Despite solidarity being firmly anchored in continental Europe, Anglo-American authors have made important contributions to its conceptual understanding. Richard Rorty’s work on solidarity is among the best known. Rorty argued against an understanding of solidarity as a universal, global concept, and against one that is based on ‘recognition of one another’s common humanity’ in any given, fixed, or general sense [2, p. 191]. Instead he proposed to see solidarity as something that has to be continuously recreated and achieved by overcoming difference between strangers, ‘by imagination, the imaginative ability to see strange people as fellow sufferers’ [2, p. xvi]. In other words, solidarity is something that requires continuous effort.

Other Anglo-American philosophers who have discussed solidarity systematically include HLA Hart [29], Max Pensky [30], and David Wiggins [31, 32]. Solidarity has also played a role in the debate between liberal philosophers (most prominently, John Rawls), and those who have been criticising some of the tenets of liberal political philosophy since the 1970s. The latter group, which includes Charles Taylor, Michael Sandel, and Michael Walzer, has been termed ‘communitarians’ (a label that some of the proponents of this group have always rejected). The latter debate, in turn, underlies the work of communitarian philosophers of medicine, such as Richard Tauber or Daniel Callahan, who stress the significance of group solidarity to balance out the perceived overemphasis on individualism in much of modern medicine and medical ethics [33–35]. Recently, the potential of solidarity to aide the implementation of universal concepts such as human rights, and its role as a foundational concept in public health, are being discussed, rather controversially, by a diverse group of Anglo-American authors (e.g., [4, 36–39]).

In addition to these discussions in moral and political philosophy, solidarity has also played a role in Anglo-American contributions by social and political scientists, social historians, economists, and others [40–47]. It must be stressed, however, that all these fields have seen an increase in works on solidarity towards the end of the 20th and particularly the beginning of the 21st century. Overall, and if considered against the attention that concepts such as justice, equality, etc. have commanded in the 20th century, solidarity has played a marginal role in Anglo-American thought, and very little if any role as a guide for practice and policy. It is noteworthy also that solidarity was not appealed to in support of the Affordable Care Act introduced in 2010. Only very recently has it started to step out of the dark, and is gaining more interest and traction.

## A fresh look at solidarity in the 21st century

In a report commissioned by the Nuffield Council on Bioethics [48], and in response to the different and often conflicting understandings of solidarity in the literature, the authors of this article have developed a working definition of solidarity. It is based on an extensive analysis of earlier understandings of solidarity. Since the first iteration of our own working definition of solidarity in 2011, we have expanded and revised it on several occasions following helpful feedback from colleagues (e.g., [39, 49, 50]) and insights obtained from applying it to practical contexts [51–53]. In its most elementary form, we understand solidarity as *an enacted commitment to carry the ‘costs’* (*financial, social, emotional, and other contributions*) *of assisting others with whom a person or persons recognise similarity in a relevant respect* [54].

Six qualifiers are in place. First, we understand solidarity first and foremost as a *practice*, that is, as purposeful engagement with the world [55, p. 4]. Solidarity is thus something that is enacted, not merely felt or thought; it is not an abstract concept. This means that analyses of solidarity need to pay careful attention to the context of a specific situation, including the policies, relations, and related practices that enable solidaristic practice.

Second, by ‘*costs*’, we refer to any contribution (e.g., in terms of time, effort, emotional investments, or money) that people make to assist others. Small things, such as giving up our own comfort to offer somebody a seat on the bus, count as ‘costs’ as much as big things, such as donating an organ or paying into a solidaristic healthcare system. That people accept such costs for the sake of helping others when practicing solidarity does not mean that they cannot, at the same time, also benefit themselves. It is only when self-interest is the main motivation that we do not consider the resulting practice as solidaristic.

Third, according to our definition, people practice solidarity with others with whom they recognise *similarity in a relevant respect*. This requirement is met if a person (or persons) considers herself to have something in common with the others who matter *in a specific situation*. Two things are important to note here. One is that the context of a specific practical situation determines the criteria of relevance. In a situation where a person offers to help a colleague study for a much feared exam because she knows what it is like to be anxious prior to an exam, this anxiety is the similarity in the relevant respect that gives rise to her solidaristic practice. That the two people in our example are different in many other respects—e.g., in religious or political beliefs—is irrelevant for the practice of solidarity in this situation. Thus, our understanding of solidarity does not assume that people are all essentially similar and that differences do not matter. On the contrary, solidaristic practice regularly takes place in a context of stark difference between persons; it takes place *despite* these differences. But it is the relevant similarity that people perceive between themselves and others that gives rise to the solidaristic action (and that we mean when we speak of a symmetrical relationship at the moment of practicing solidarity).

The other important thing to note is that the relevant similarity that a person perceives between herself and another is entirely subjective; we do not believe that people have objective characteristics that are, or should be, recognised as things that bind people together. To stick with our aforementioned example, a person’s offer to help her anxious colleague could just as well not recognise the relevant similarity and not enact solidarity if, for example, she were to focus on what sets her apart from her colleague. The colleague may belong to a social elite that she despises, or perhaps the colleague is very religious, while Lin is staunchly atheist. What people recognise as a relevant similarity is subjective, but not arbitrary. Political discourses can make it more or less likely that individuals perceive similarities rather than differences. A political discourse that foregrounds how much additional public money is spent specifically on immigrants fosters a situation in which people start to see immigrants as different from them. All that they share in common moves to the background. In contrast, a political discourse that highlights how hard most immigrants work to build a decent living and to contribute to society in their new homes is conducive to people focusing on the similarities.

Fourth, in order to focus discussion on the level on which solidaristic practices take place—ranging from interpersonal to being part of the ‘fabric of society’—we distinguish between *three tiers of solidarity*. The first tier of solidarity refers to solidaristic practice between individual people. Our vignette of a student providing help to her colleague is an example of solidarity at this level. If practices of solidarity between people are so common in a given context or community that they become something ‘normal’, then we speak of second tier solidarity. If the values or principles enacted and emerging through group or community-based practices in this manner solidify further and become written into *contractual, legal, or administrative norms,* then we have instances of third tier solidarity. The relationship between the three tiers is such that solidaristic norms and provisions at the third tier have often emerged out of initially more informal practices of solidarity at the interpersonal (first tier) or communal or group level (second tier). But of course, not all inter-personal practices at the first tier actually solidify into practices and norms at the second and third tiers. In societies where discursive, political, and economic structures emphasise and foster concern for others, and where the wellbeing of individuals is seen as closely connected to the wellbeing of others, these structures provide the ‘glue’ between the three tiers. They provide the background conditions for solidarity to grow and proliferate. If such structures are not in place, then even though solidaristic practice at each of the tiers may emerge, they would hang ‘in thin air’. In such a situation solidaristic practices would be unlikely to proliferate, flourish, and solidify.

Fifth, many authors see solidarity as something that is directed mostly towards *vulnerable* people [56, 57]. The role vulnerability plays in our framework hinges on the notion of similarity. One of the core features of solidarity is that it foregrounds similarities between people, and that these similarities are determined by the specifics of a concrete practice or setting. As argued above, while two people, or members of different groups, may be very different in many respects, one single similarity, if it *matters enough* in a concrete situation, can give rise to solidarity. If we have, for example, experienced a serious illness earlier in our lives, we may donate money and provide other means of support to others who suffer from the same disease, even if we have never met them in person and they live on the other side of the world. The shared experience of living with this illness becomes a defining moment in our practice of solidarity, even though there may be nothing else that we see ourselves having in common with the people whom we are helping. The shared experience is what makes us assist these people. This assistance, in turn, is typically something that affirms, helps, or supports others, and not something that harms them. It is in this sense that vulnerability matters. Despite the fact that what motivates our action is a commonality that we consider ourselves sharing with others, in the concrete situation in which solidarity is enacted, it is they, and not us, who are in need of support. That they are vulnerable and in need of support in this moment thus determines the direction of the support in the concrete instance of solidarity. But it does not change the fact that commonality between us, and not our differences, gives rise to our action.

Sixth, many authors see *reciprocity* as closely related to, or even synonymous with, solidarity. Indeed, the relationship between these concepts is such that for solidarity to grow and institutionalise, a certain level of reciprocity is required. But how exactly does reciprocity support the practice and institutionalisation of solidarity? As mentioned above, if reciprocity is the decisive or even the only motivation for a practice, then this practice should be called solidaristic. But particularly at higher levels of institutionalisation, indirect reciprocity can play an important role for stabilising solidaristic arrangements, and overall attitudes of reciprocity will help maintain and enliven solidaristic policies and laws.

This understanding of solidarity shares elements with definitions from other authors, and includes some features that resonate with the historical understandings briefly introduced in our first section above. However, at the same time, this conceptualization serves to divest the concept of solidarity of some of the more overtly partisan connotations it has been associated with in the past. In our understanding, solidarity is not an intrinsic good or a ‘leftist’ ideal; neither is it tied to or emergent from a particular understanding of a nation state or the common good. The normative value of the concept as such—independent of its concrete instantiations—is that it appreciates and facilitates non-calculating mutual support. As such, and possibly in contrast to more particularistic understandings of solidarity, this concept lends itself well to guiding policy and practice to address issues with global relevance.

In the next section, we provide an example of how solidarity can guide policy in an area that is of increasing relevance locally and globally, namely, the mitigation of harms emerging from the use of personal data. We argue that this solidarity-based governance framework can be fruitfully implemented on both sides of the Atlantic, irrespective of the differences in doctrinal and cultural stances on the political value of solidarity.

## Solidarity in practice: the establishment of harm mitigation funds for personal data use

Digital data and ‘big data’ analyses and epistemologies are playing an increasingly important role in medical practice and medical research. Whether we call it ‘personalised’ or ‘precision’ medicine, or whether we are skeptical of any of these concepts, it is clear that the trend in medicine is towards data-driven research and practice [58–60]. Moreover, data will increasingly come not only from the usual places, e.g., medical research and the clinic, but also from others such as public archives, people’s personal devices, etc. This is especially so as combining and linking different datasets has become much easier.

How we govern data use is thus a more important question than it has ever been. Policy makers are recognising this. In the European Union, a new General Data Protection Regulation (GDPR) will come into effect in 2018. It will expand people’s right to control who collects and uses data, including the right to have personal data deleted in some circumstances. Such recognition is important, but, we argue, more is needed. Many data governance frameworks rely on distinctions that no longer work in the digital era. First, in a time when data and datasets can be copied, linked, and shared more easily than ever, the distinction between identified and anonymised data is no longer firm; almost any dataset is potentially identifiable when linked with sufficient numbers and types of other datasets. Similarly, data most likely to harm data subjects if put to unauthorised use were previously categorized separately as ‘sensitive’ data. Typical examples were medical information, information on sexual orientation, and religious or political affiliations. Today, virtually any dataset can harm data subjects and others if used against them. In the case of consumer scoring, for example, seemingly innocuous pieces of information about a person can lead to the person being profiled as high-risk of defaulting on a mortgage or overusing emergency rooms if the analysis of large datasets has shown that these characteristics correlate with higher risk at the aggregate level [61, 62]. Also, because even the most innocuous piece of information—such as where people shop or in what postcode they live—can be used in health-related contexts (e.g., to mark people who are more likely to be ‘expensive’ patients), any dataset is potentially health-relevant. In short, any and all data are now potentially identifiable, health-related, and sensitive.

Given these developments, should the use and integration of personal data in biomedicine be even more strictly regulated? We argue that this would be the wrong approach. There are many benefits to increased data use and analysis in biomedicine. However, it would be wrong to believe that one can carry out a comprehensive risk assessment *a priori*. It is the use of data for certain purposes, not the nature of the data, that makes them more (or less) likely to harm data subjects. We believe that a solidarity-based perspective can help to develop a governance framework that responds to this challenge, while allowing the kinds of data usage we want to support to further the improvement of clinical care and biomedical research. At the same time, it would help to ensure that those who allow their data to be used for public benefit are not left alone when something goes wrong. Such a governance framework would mandate a differentiation between data use in the public interest and data use that is not in the public interest. Building upon and expanding the definition of public value expressed by participants in a large Wellcome Trust study [63, p. 9], we consider data use to be in the public interest if it will plausibly have clear benefits for many patients, society as a whole, or future generations, and no person or group will experience significant harm. Moreover, public interest is more pronounced if the benefits are likely to materialise for underprivileged groups than for privileged people, due to the overall lower baseline and potential size of impact (underprivileged groups usually benefit more from public action; we leave out cases where this might not be so). An example for data use in the public interest is the analysis of clinical datasets to see the commonalities shared by patients who recover particularly fast after a specific surgery. An example of data use that is not in the public interest would be targeted marketing.

It is important to note that we are talking about data *use* here, not data collection; the question of public interest is not a property of the data themselves, but of the context and purpose of their use. In practice, a solidarity-based approach to data use governance entails the following steps. First, the risk for unauthorised^3^ re-identification for all sets of data, regardless of the purpose for which they are collected and used, should be decreased where meaningfully and reasonably possible (e.g., by using privacy-by-design or privacy-by-default models). As a second step, the exchange and analysis of data for purposes that are in the public interest should be made easier. This would mean making regular use of instruments such as broad consent or ‘consent to be governed’ [64], in combination with oversight by independent governance boards. It would also mean bidding farewell to the illusion that greater *individual* control over data use at every step of the process is the best way to address the ethics of digital data use.

In addition to these steps, we also propose the establishment of harm mitigation funds [40, 52, 54]. A harm mitigation fund would be an institution governed by people who are independent of the organisations using data (the ‘data controller’, in the nomenclature of the GDPR) and who review appeals from people who claim to have been harmed by data use. Harm mitigation funds could make positive decisions on appeal even when no laws or rules are broken.

It is important to note that harm mitigation funds are not intended to replace existing legal instruments. They would exist to complement, not replace, legal provisions, and would not affect any statutory rights of individuals. Compared with legal instruments, harm mitigation would be a more flexible instrument, designed to accommodate the specific circumstances in which harm resulting from data use has, or is believed to have, occurred. This does not mean, however, that decisions made by harm mitigation funds would be arbitrary. A firm set of questions and indicators would guide (but not determine) the assessment. These questions and indicators would be made transparent and easily accessible. Harm mitigation policies would also include categories of eligible harm, indicators to assess causality, criteria to assess severity of harm, clear mechanisms of compensation or other responses (e.g., issuing of public apologies, data user feedback systems to prevent the recurrence of harms, etc.), and most importantly, transparent rules on the qualifications of people serving on governance boards, on term limits, and on resolving conflicts of interest. To the best of our knowledge, such instruments have not yet been implemented. There are, however, precursers of this type of governance, such as German ‘registered societies’ (*eingetragener Verein*).

Moreover, in addition to providing financial support to people who are harmed by data use, harm mitigation funds would provide feedback to data users (and where appropriate, regulators) on how systems and governance frameworks could be improved. The funds for harm mitigation could come from a small portion of a project or institution’s budget or from taxation. Harm mitigation funds could be established at the level of individual institutions or organisations, or they could cover a region or nation. Importantly, harm mitigation funds would hear appeals not only from people who feel they are harmed by the use of *their own* data but also by use of other people’s data.^4^

## Conclusion

The establishment of harm mitigation funds—if enacted in the public interest, introduced transparently, and with the requirements of general risk minimisation described above—deviates from the current emphasis on addressing risk by expanding individuals’ control over their data at every step of the way, an approach that we believe is ineffective for avoiding or mitigating harm at the personal and societal level. Our approach, in contrast, entails the acceptance of the impossibility of eliminating all the risks of modern data usage. A solidarity-based framework accepts the existence of risks, and assumes that many are willing to take those risks to support others with whom they recognise a relevant similarity. Harm mitigation funds help to ensure that people who accept such risks and are harmed as a result have appropriate support. To our minds, this responds to the need to enable solidaristic, pro-social practices and policies to be more explicitly recognised and fostered in current health policy and beyond.

Additionally, as introduced above, the idea of solidarity underlying harm mitigation funds is well suited for application to issues that are increasingly regarded as globally relevant. Usage of (digital) data in various forms is rapidly on the rise, and by its very nature, does not conform to national borders. This is also the case, increasingly, in other areas of health care, medicine, and research, due to the ever-increasing mobility and interconnectedness of patients, expertise, and knowledge. To respond to the resulting challenges, ‘localised’ ideas and approaches, such as distinctly European or Anglo-American principles, values, frameworks, etc., will likely have to be complemented by those that transcend locality, or be reframed with a view to more transnational application. Solidarity, notwithstanding its European heritage, is a concept that has a wide range of application from the local and particular to the broad and global [66]. Therefore, we believe that regulatory elements based on solidarity, such as the harm mitigation funds introduced in this article, can usefully be implemented on both sides of the Atlantic.

## Footnotes

1 See also [26, 27].

2 Tomasini cites Manuel Castells [28] here.

3 Our emphasis here is on the word unauthorised; in some cases re-identification is not problematic or even desired (e.g., people who make data available on platforms such as DNA.land or OpenHumans). Not every instance of re-identification of de-identified data is unauthorised, and not even every unauthorised re-identification leads to harm.

4 See, e.g., Mark Taylor’s work on the rights of secondary data subjects [65].
